# Orexin-A in Patients With Lewy Body Disease: A Systematic Review and Meta-Analysis

**DOI:** 10.3389/fendo.2021.765701

**Published:** 2021-11-17

**Authors:** Jinghuan Gan, Zhichao Chen, Jiuyan Han, Lingyun Ma, Shuai Liu, Xiao-Dan Wang, Yong Ji

**Affiliations:** ^1^ Department of Neurology, Beijing Tiantan Hospital, Capital Medical University, Beijing, China; ^2^ Department of Neurology, Tianjin Huanhu Hospital, Tianjin, China

**Keywords:** orexin-A (hypocretin-1), Lewy body disease, Parkinson’s disease, excessive daytime sleepiness, systematic review, meta-analysis

## Abstract

**Background:**

Abnormal orexin-A levels in cerebrospinal fluid (CSF) have been identified in Alzheimer’s disease (AD) and other neurodegenerative diseases. However, few studies have focused on Lewy body disease (LBD) and often with debatable outcomes. Thus, we performed this systematic review and meta-analysis to investigate orexin-A levels in LBD by incorporating data from different studies.

**Methods:**

We gathered studies comparing orexin-A levels in patients with LBD and controls (including healthy controls and other dementia subtypes). In the initial search, 117 relevant articles were identified. After a selection process, seven studies, conducted in Japan, USA, Spain, Switzerland, France, Italy, and Netherlands, were chosen.

**Results:**

In total, 179 patients with LBD and 253 controls were included. Patients with LBD had significantly lower mean orexin-A CSF levels when compared with patients with AD [standard mean difference (SMD): −0.35, 95% confidence interval (CI): −0.70 to −0.00, Z = 1.96, P = 0.05], whereas mean orexin-A levels were significantly higher when compared with patients with frontotemporal lobar degeneration (FTLD) (SMD: 0.61, 95% CI: 0.23–0.99, Z = 3.12, P = 0.002). Orexin-A CSF levels in LBD patients were approximately equal to levels in healthy elderly individuals, whereas they were significantly decreased in LBD patients with excessive daytime sleepiness (EDS) (SMD: -0.15, 95% CI: -0.59 to 0.29, Z = 0.67, P = 0.50).

**Conclusions:**

We showed that orexin-A levels in patients with LBD were not very different from those in normal elderly individuals, whereas they were lower than those in AD patients and higher than those in FTLD patients. The influence of hypersomnia on orexin-A levels should be carefully interpreted.

**Systematic Review Registration:**

https://www.crd.york.ac.uk/prospero/, identifier CRD42021265900.

## Introduction

Lewy body disease (LBD) is characterized by the presence of Lewy bodies (LBs) and Lewy neurites, with senile plaque (SP) and neurofibrillary tangle (NFT) deposition (1). LBD comprises a diagnostic spectrum that includes Parkinson’s disease (PD), PD with dementia (PDD), and dementia with Lewy bodies (DLB). Currently, DLB and PDD are differentiated based on the “one-year rule,” which accounts for the relationship between parkinsonism symptoms and dementia.

Sleep disorders, neuropsychiatric symptoms, and parkinsonism symptoms are common clinical characteristics of LBD, given their high prevalence and association with poorer health-related outcomes (2). Although the exact pathophysiology is poorly understood, the orexinergic system has been implicated in some regulatory mechanisms. The hypothalamic neurotransmitter, orexin-A (hypocretin-1), is believed to play a crucial role in the orexinergic system; it contributes to the regulation of the sleep–wake cycle by increasing arousal levels and maintaining wakefulness and maintaining memory and spatial cognition functions (3, 4). Also, orexin-A interacts with multiple areas in the brain stem and other neurotransmitter systems, including dopamine (5).

Neuropathological studies have identified a 25% reduction in hypothalamic hypocretin neurons and LBs in PD patients at early stage I disease and a 62% reduction in advanced PD patients (6). Several clinical studies have investigated orexin-A levels in cerebrospinal fluid (CSF) in patients with LBD; however, study findings and associations with sleep disturbance remain unclear (7). Previous studies indicated that narcolepsy was linked with reduced orexin-A levels (8), and a correlation was identified between neocortical orexin-A levels and hypersomnolence in patients with DLB (9). Orexinergic overexpression may have important roles in the manifestation of rapid-eye-movement sleep behavior disorders (RBDs) in DLB; previous evidence described a significant positive correlation between RBD severity and CSF orexin-A levels (10).

PD is the second most common neurodegenerative disease and performs complex clinical manifestations. PDD can also occur in many patients with PD up to 90% in their long-term, with illness progression (11). The incidence of DLB, which is the second most common neurodegenerative dementia, is approximately 3.5 per 100,000 person-years (12) and accounts for 10%–20% of all dementias (13, 14). The prevalence of sleep disturbance in LBD is 44%–55% (15) and is more prominent than in Alzheimer’s disease (AD) patients. While little research has been conducted on orexin-A levels in LBD, there is ongoing debate in the literature regarding orexin-A associations with LBD clinical manifestations (6, 9, 16, 17).

To address this knowledge gap, we designed this systematic review and meta-analysis to summarize orexin-A levels in CSF in LBD and explore associations between these levels and sleep disturbance. We hypothesized that lower CSF orexin-A levels in LBD could be a reliable biomarker to discriminate between LBD and AD/frontotemporal lobar degeneration (FTLD).

## Methods

### Data Sources and Literature Searches

This systematic review and meta-analysis were prepared using Preferred Reporting Items for Systematic reviews and Meta-Analyses (PRISMA) guidelines (18). We conducted an electronic literature search using PubMed, Embase, Web of Science, Cochrane library, Wanfang Data, China National Knowledge Infrastructure (CNKI), and China Biology Medicine Disc (CBM) databases from the earliest available date to June 1, 2021. The literature search was conducted using the following search terms/keywords, without limits: (“Lewy body disease” OR “Diffuse Lewy Body Disease” OR “Lewy Body Dementia” OR “Cortical Lewy Body Disease” OR “Lewy Body Disease, Cortical” OR “Lewy Body Type Senile Dementia” OR “Lewy Body Disease, Diffuse” OR “Dementia, Lewy Body”) AND (“Orexin” OR “Hypocretins” OR “Hypocretin” OR “Orexin” OR “Orexin-B” OR “Orexin B” OR “Hypocretin-2” OR “Hypocretin 2” OR “Orexin-A” OR “Orexin A” OR “Hypocretin-1” OR “Hypocretin 1”). The search was not restricted by region, race, or age. In addition, bibliographies in retrieved papers and reviews were manually searched to capture eligible studies. Two authors (JG and ZC) independently performed literature searches and screened all titles, abstracts, and full texts of eligible studies. Differences in opinion were discussed with the corresponding author (YJ) and resolved by mutual consensus.

**Figure 1:**
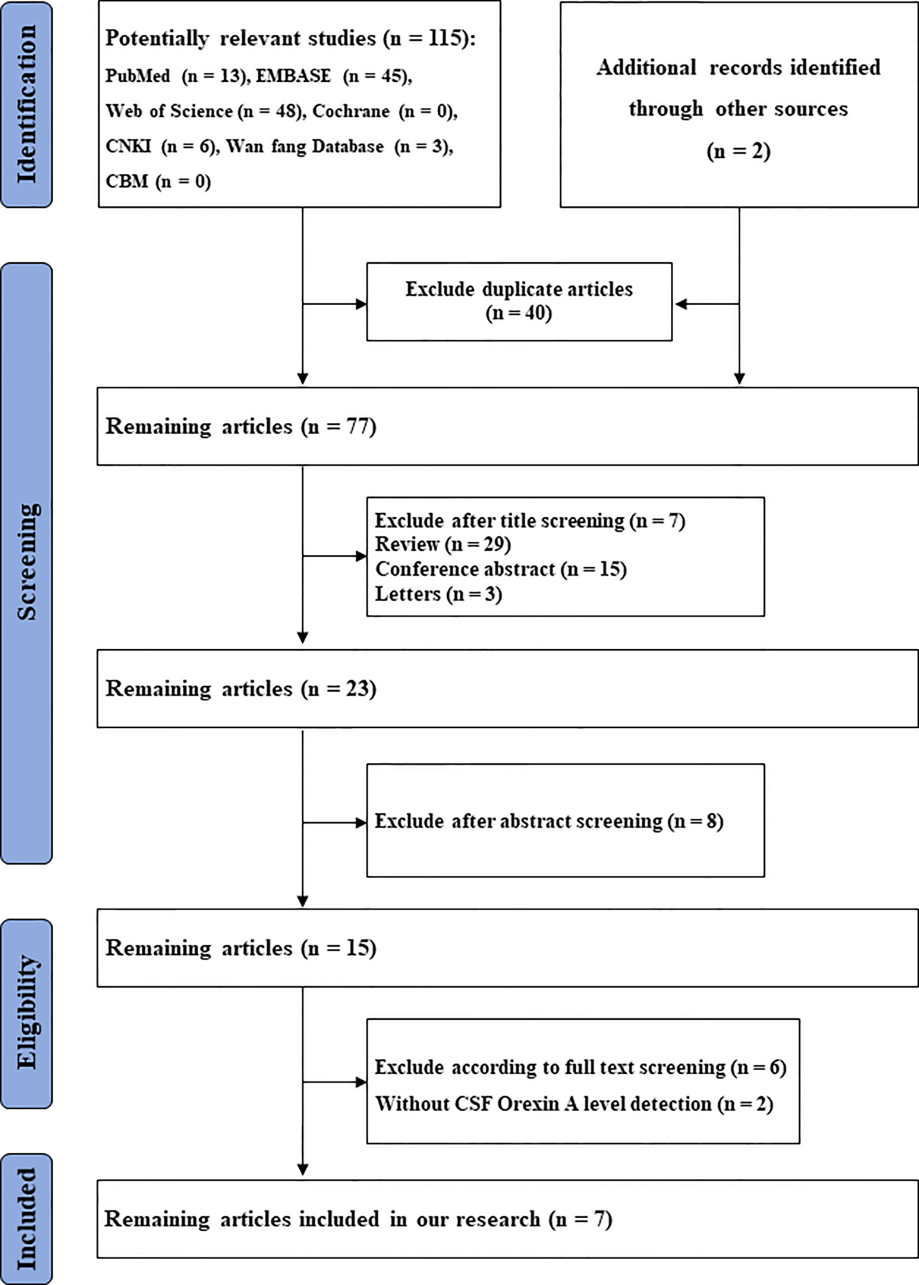
Flowchart of study selection. CSF, cerebrospinal fluid.

A flowchart, based on PRISMA guidelines, of search and screening strategies and eligibility criteria is shown (**Figure 1**).

### Inclusion and Exclusion Criteria

Our analyses included studies that met the following criteria: 1) The primary eligibility criterion was a comparison of CSF orexin-A levels in patients with LBD and healthy controls/any other dementia subtypes; 2) Studies that provided a complete definition of participants. The diagnosis of different types of dementia/healthy controls was defined using clinical evaluations and neuropsychological tests: AD was defined by the Diagnostic and Statistical Manual of Mental Disorders, Fourth Edition; the International Statistical Classification of Diseases and Related Health Problems, 10th Revision, criteria of the National Institute of Neurological Communicative Diseases and Stroke, and the Alzheimer’s Disease and Related Disorders Association. PDD was defined by the United Kingdom Parkinson’s Disease Society Brain Bank. DLB was defined by DLB consensus criteria. FTLD was defined by international FTLD consensus criteria; and 3) Orexin-A levels were measured in CSF samples, with sufficient data (pg/dl).

Similarly, studies were excluded if they met any of the following criteria: 1) no available CSF orexin-A data; 2) the publication was a letter, review, meta-analysis, or animal study; and/or 3) the orexin-A detection method differed from the guidelines.

### Statistical Analysis

Review Manager 5.3 (Nordic Cochrane Centre, Cochrane Collaboration, Copenhagen, Denmark) was used for statistical analyses and the graphical presentation of data. Since CSF orexin-A levels were continuous, the standard mean difference (SMD) was calculated and used for statistical analyses. Medians and interquartile ranges were converted to means and standard deviation (SD) in accordance with the methodology of Hozo et al. (19).

A fixed-effects model was used for the meta-analysis due to its superior statistical power over the random-effects model. The heterogeneity statistic was *I^2^* < 10%. Statistical heterogeneity among studies was investigated using a χ^2^ test, with calculated Q statistics, corresponding P values, and *I^2^* statistics. A P > 0.1 or *I^2^* < 50% value indicated no significant heterogeneity among studies. A P < 0.05 or *I^2^* > 75% indicated significant heterogeneity, and a random-effects model was performed.

Publication bias was assessed using funnel plots. Where a distribution was not roughly symmetrical, an increased risk of bias was indicated.

## Results

### Study Selection and Characteristics

**Figure 2:**
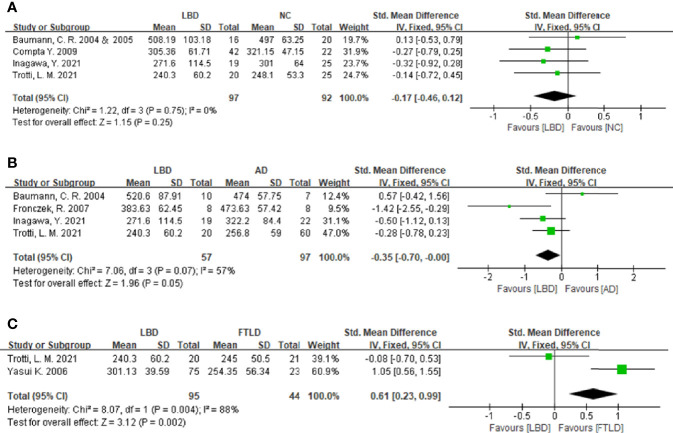
Forest plots of the CSF orexin-A levels in LBD compared with NCs/other diseases. **(A)** Forest plot of CSF orexin-A levels in LBD compared with NCs. **(B)** Forest plot of CSF orexin-A levels in LBD compared with AD. **(C)** Forest plot of CSF orexin-A levels in LBD compared with FTLD. CI, confidence interval; CSF, cerebrospinal fluid; LBD, Lewy body disease; NC, normal controls; AD, Alzheimer’s disease; FTLD, frontotemporal lobar degeneration.

In total, we identified 117 studies. After removing duplicates (n = 40), 77 studies remained. From these, 54 studies were excluded because they were reviews (n = 29), letters (n = 3), conference abstracts (n = 15), experimental animal studies or other disease investigations (n = 7). From the remaining 23 studies, eight were excluded after abstract review, six were excluded after full-text review, and two excluded due to a lack of CSF orexin-A data. The remaining seven articles, with 432 participants, 179 with LBD and 253 controls, were finally included in the meta-analysis (6, 7, 10, 16, 17, 20, 21) (**Table 1**). Two studies (7, 17) came from the same research team and had the same control group; therefore, we analyzed them together. The results are shown (**Figure 2**).

**Table 1:** Characteristics of seven case-control studies included in meta-analysis.

Study	Year	Country	Design	LBD group				Control group			
				Sample size	Gender(M/F)	Age^a^(Years)	Orexin-A level(pg/ml)	Sample size	Gender(M/F)	Age^a^(Years)	Orexin-A level(pg/ml)
Inagawa et al. (10)	2021	Japan	Case-control	19 (DLB)	10/9	81.1 ± 3.1	271.6 ± 114.5	25 (NC)	15/10	71.4 ± 11.2	301.0 ± 64
								22 (AD)	11/11	73.9 ± 8.0	322.2 ± 84.4
Trotti et al. (20)	2021	USA	Case-control	20 (DLB)	15/5	64.9 ± 9.8	240.3 ± 60.2	25 (NC)	8/17	69.6 ± 9.2	248.1 ± 53.3
								60 (AD)	31/29	65.5 ± 7.7	256.8 ± 59.0
								21 (FTLD)	14/7	66.3 ± 9.2	245.0 ± 50.5
Compta et al. (16)	2009	Spain	Case-control	21 (PD)	12/9	68.8 ± 6.9	300.99 ± 58.68	22 (NC)	10/12	70.4 ± 9	321.15 ± 47.15
				20 (PDD)	9/11	72.5 ± 7.14	309.94 ± 65.95				
Baumann et al. (7)	2004	Switzerland (n = 9) France (n = 1)	Case-control	10 (DLB)	7/3	72.4 (60–82)	521 (382–667)	20 (NC)	None	44 (17–79)	497 (350–603)
								7 (AD)	None	72 (49–84)	474 (333–564)
Baumann et al. (17)	2005	Switzerland (n = 2) Italy (n = 4)	Case-control	6 (PD)	4/2	71 ± 8.81^b^	487.5 ± 131.16^c^	20 (NC)	None	44 (17–79)	497 (350–603)
										
Yasui et al. (21)	2006	Japan	Case-control	62 (PD)	23/39	69.5 ± 9.7	302 ± 38	16 (PSP)	11/5	72.0 ± 5.9	258 ± 37
				13 (DLB)	7/6	75.7 ± 6.3	297 ± 48	7 (CBD)	3/4	71.4 ± 8.1	246 ± 90
Fronczek et al. (6)	2007	Netherlands	Case-control	8 (PD)	5/3	76.88 ± 11.67 ^d^	383.63 ± 62.45^e^	8 (NC)	5/3	74 ± 8.19^f^	473.63 ± 57.42^g^

M/F, male/female; NC, normal controls; AD, Alzheimer’s disease; CSF, cerebrospinal fluid; DLB, dementia with Lewy bodies; PD, Parkinson’s disease; PDD, Parkinson’s disease dementia; FTLD, frontotemporal lobar degeneration; PSP, progressive supranuclear palsy; CBD, corticobasal degeneration.

a The ages were displayed as mean (± standard deviation) or median (range).

b The ages of six patients in this study were 70, 78, 73, 74, 77, and 54 years old, respectively.

c The CSF orexin-A levels of six patients in this study were 591, 380, 307, 654, 537, and 454 pg/ml, respectively.

d The ages of eight patients in this study were 56, 62, 77, 80, 81, 86, 86, and 87 years old, respectively.

e The CSF orexin-A levels of eight patients in this study were 404, 327, 327, 331, 377, 463, 354, and 486 pg/ml, respectively.

f The ages of eight normal elderly in this study were 56, 69, 77, 78, 82, 74, 78, and 78 years old, respectively.

g The CSF orexin-A levels of eight normal elderly in this study were 554, 468, 492, 472, 368, 422, 495, and 518 pg/ml, respectively.

### Cerebrospinal Fluid Orexin-A Levels in Lewy Body Disease *vs*. Normal Controls

To compare CSF orexin-A levels in LBD patients against NCs, five studies ( 7, 10, 16, 17, 20) were included (97 LBD patients *vs*. 92 NCs). Studies showed low heterogeneity (*I^2^* = 0%, P = 0.75), so a fixed-effects model was used to calculate the pooled SMD. A forest plot demonstrated slightly lower mean CSF orexin-A levels in LBD when compared with NCs; however, this was not statistically significant (SMD: -0.17, 95% CI: −0.46 to 0.12, Z = 1.15, P = 0.25) (**Figure 2A**).

### Cerebrospinal Fluid Orexin-A Levels in Lewy Body Disease *vs*. Alzheimer’s Disease

Four studies were included in this section of the meta-analysis. LBD and AD patient numbers were 57 and 97, respectively. Study heterogeneity was low (P = 0.07, *I^2^* = 57%), so a fixed-effects model was used to calculate the pooled SMD. A forest plot demonstrated significantly lower mean CSF orexin-A levels in LBD patients when compared with AD patients (SMD: −0.35, 95% CI: −0.70 to −0.00, Z = 1.96, P = 0.05) (**Figure 2B**).

### Cerebrospinal Fluid Orexin-A Levels in Lewy Body Disease *vs*. Frontotemporal Lobar Degeneration

Sixteen progressive supranuclear palsy (PSP) and 7 corticobasal degeneration (CBD) patients were included as controls in the study by Yasui et al. (21). Since PSP and CBD are untypical FTLD subtypes (22), two studies (20, 21) were included in the meta-analysis. LBD and FTLD patients were 95 and 44, respectively. Study heterogeneity was high (P = 0.004, *I^2^* = 88%). A forest plot demonstrated significantly higher mean CSF orexin-A levels in LBD patients when compared with FTLD patients (SMD: 0.61, 95% CI: 0.23–0.99, Z = 3.12, P = 0.002) (**Figure 2C**).

### Cerebrospinal Fluid Orexin-A Levels in Lewy Body Disease With Excessive Daytime Sleepiness *vs*. Normal Controls

**Figure 3:**
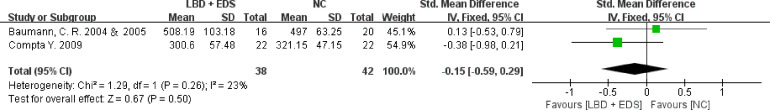
Forest plots of the CSF orexin-A levels in LBD with EDS compared with NCs. CSF, cerebrospinal fluid; LBD, Lewy body disease; NCs, normal controls; EDS, excessive daytime sleepiness; CI, confidence interval.

A subgroup analysis was conducted, and pooled SMD estimates were stratified by EDS. CSF orexin-A levels in LBD patients with EDS were lower than those in NCs but were not statistically significant (SMD: -0.15, 95% CI: -0.59 to 0.29, Z = 0.67, P = 0.50) (**Figure 3**).

### Publication Bias

**Figure 4:**
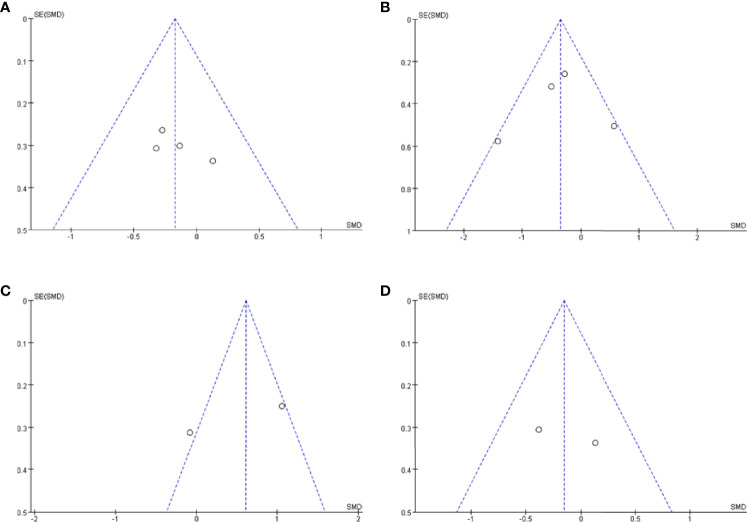
Funnel plot of CSF orexin-A levels in LBD compared with NCs/other diseases. **(A)** Funnel plot of CSF orexin-A levels in LBD compared with NC. **(B)** Funnel plot of CSF orexin-A levels in LBD compared with AD. **(C)** Funnel plot of CSF orexin-A levels in LBD compared with FTLD. **(D)** Funnel plot of CSF orexin-A levels in LBD with EDS compared with NCs. CSF, cerebrospinal fluid; LBD, Lewy body disease; NCs, normal controls; AD, Alzheimer’s disease; FTLD, frontotemporal lobar degeneration; EDS, excessive daytime sleepiness; SMD, standardized mean difference.

Publication bias was assessed using a visual inspection of funnel plots for each analysis. All showed relatively symmetrical distributions indicating a low risk of publication bias (**Figures 4A–D**).

## Discussion

We demonstrated the potential role of CSF orexin-A levels in the differential diagnosis of LBD and other diseases. In this meta-analysis, mean CSF orexin-A levels were significantly lower in LBD patients when compared with those in AD patients and higher when compared with those in FTLD patients. Mean CSF orexin-A levels in LBD patients were lower than NCs but lacked statistical significance. Thus, CSF orexin-A levels could be used as a reliable biomarker to discriminate between LBD and AD/FTLD and be used to support clinical and neuroimaging findings to increase the accuracy of clinical diagnoses for these conditions.

Orexin consists of orexin-A and orexin-B and are neuropeptides involved in the regulation of the sleep–wake cycle, learning and memory, motor control, and autonomic nervous function by its wide range of intracranial projections and neurotransmitters (23, 24). CSF orexin-A levels have been extensively studied in sleep disturbance studies (e.g., narcolepsy, insomnia, and RBD) and AD. Equally, several small-sample studies on LBD manifestations have also been published.

### Cerebrospinal Fluid Orexin-A Levels in Lewy Body Disease Patients and Normal Controls

We demonstrated that mean CSF orexin-A levels in LBD were similar to levels in NCs. DLB and PD are the two main forms of LBD and share common clinical and neuropathological traits (25). Despite previous studies showing reductions in neocortical orexin-A levels in patients with DLB (9) and significantly decreased orexinergic neurons in patients with parkinsonian traits and PD animal models, the mechanisms underlying these reductions remain poorly understood. Perhaps the loss of orexin neurons is insufficient to cause significant changes in CSF orexin-A levels. A previous animal study reported that 15% damage to orexinergic neurons did not affect CSF orexin levels, whereas more than 70% damage led to a 50% decline in these levels (26). A recent study on orexin-A plasma levels during PD progression showed that levels were increased in early and medium disease stages and were decreased at advanced PD stages. Orexin-A levels in PD patients were significantly negatively correlated with disease duration and the Movement Disorder Society Unified Parkinson's Disease Rating Scale (MDS-UPDRS) Part III scores (27). These findings may explain how plasma orexin-A levels occur during different PD stages. However, apart from the studies included in this analysis (6, 7), Wennstrom et al. (28) and Lessig et al. (9) reported that orexin-A levels in DLB were significantly lower than those in NCs. While our results are controversial, discrepancies may have arisen due to differences in patient numbers, average patient ages, and cognitive functions.

### Cerebrospinal Fluid Orexin-A Levels in Lewy Body Disease and Alzheimer’s Disease Patients

Consistent with the animal and human literature (29, 30), we generally concluded that patients with LBD have lower orexin-A levels when compared with AD patients, potentially suggesting different orexin-A roles in AD and LBD. Sleep disturbance is a common feature of many neurodegenerative diseases, and critically, orexin-A is closely associated with sleep disturbance.

Previous investigations suggested that orexinergic signaling overexpression primarily altered the sleep–wake cycle to promote wakefulness and secondarily induced Beta-amyloid (Aβ) accumulation and Tau-mediated neurodegeneration (31, 32). Both human and animal models suggested that orexin-A may play a role in AD pathogenesis. Aβ levels were significantly increased during acute or long-term sleep distribution and orexin infusion, whereas levels were decreased upon infusion with a dual orexin receptor antagonist. An observational study of 17 AD patients showed that orexin-A was significantly related to Tau (r = 0.463, P < 0.001) and phosphorylated Tau (r = 0.630, P < 0.0001) (33, 34), and that orexin receptor downregulation in the hippocampus, especially the cornu ammonis, was caused by Aβ formation and Tau hyperphosphorylation (32). Therefore, we speculate that disease-specific alterations (orexinergic system damage Aβ modulation in narcolepsy and amyloid pathology in AD) induce the loss of reciprocal Aβ modulation between orexin and amyloid-42 CSF levels (32). Perturbations in both orexin signaling and the sleep–wake cycle may induce adverse effects on maintaining sleep and Aβ dynamics.

A previous study indicated that hypersomnia was more prevalent in LBD than AD (35), in agreement with sleepiness traits using LBD diagnostic criteria (2). Recent evidence has suggested that hypersomnia is associated with LBD (36); increased immunocompetence of orexin neurons and decreased CSF orexin-A levels may account for this finding (37). When orexin neurons are deficient, the balance between sleep and wakefulness is disturbed. Interactions between sleep-active neurons and monoamine-induced wakefulness were weakened in the ventrolateral preoptic nucleus, which reduced balance stability between sleep and wakefulness, leading to narcolepsy. Thus, hypersomnia was identified in animal models bearing orexin receptor gene mutations or receptor knockout phenotypes. In other research involving LBD (including DLB, PD, or PDD) and AD, CSF orexin-A levels in LBD patients were significantly lower than those in the AD group (9, 28). In addition, significant correlations were identified between CSF orexin-A levels and sleep disturbance severity (10) or the scores of Epworth sleepiness scale (ESS), a scale used to evaluate EDS/hypersomnia (7), after calculation. These findings on the connection between AD and LBD remain controversial. In our study, we observed no significant decrease in orexin-A levels in LBD patients when compared with AD groups in selected studies (7, 10, 20). Similarly, we observed no statistically significant differences in orexin-A plasma levels between patients with AD and patients with PD (38).

CSF orexin-A reductions (7, 10, 21) and immunoreactivity in DLB (9) are both correlated with hypersomnia and α-synuclein accumulation, as proposed for PD (6, 21) and an experimental animal model (39). Also, previous postmortem studies revealed the existence of LBs in the hypothalamus of PD patients (40). A recent study also identified α-synuclein accumulation in orexinergic neurons in an A53T mouse model of PD (41). Lessig et al. (9) demonstrated a significant negative correlation (r = −0.447, P < 0.05) between orexin-A levels and α-synuclein aggregation in DLB patients, consistent with previous PD studies showing α-synuclein accumulation in hypocretin-containing neurons in the hypothalamus (6, 42). Unfortunately, few studies have investigated α-synuclein and orexin-A levels in humans; therefore, further studies are required.

### Cerebrospinal Fluid Orexin-A Levels in Lewy Body Disease and Frontotemporal Lobar Degeneration Patients

FTLD is a clinically and pathologically diverse group of younger-onset diseases, with patients reportedly developing hypersomnia, insomnia, and other sleep-related symptoms (38, 43, 44). Sleep disturbance in FTLD forms part of a much broader spectrum of homeostatic and related behavioral alterations; the plasma or CSF orexin-A reductions were correlated with hypersomnia (38, 44).

Several small-scale studies compared orexin-A levels between LBD and FTLD patients and reported that reduced levels were related to poor-quality sleep or hypersomnia in FTLD; however, these data were inconclusive (21, 38), as other studies identified no alterations in orexin-A levels (20, 44). Liguori et al. (44) observed no significant differences in CSF orexin-A levels between FTLD patients and controls, whereas their Spearman Rank Order data identified a significant negative correlation between CSF orexin-A levels and ESS scores (r = -0.91, P < 0.0001) in the global FTLD population. A group of 10 consecutive patients with FTLD showed statistically lower orexin-A plasma levels (106 ± 29 pg/ml) than patients with AD (154 ± 74 pg/ml) and patients with PD (135 ± 92 pg/ml) (38). In this cohort, two FTLD patients underwent polysomnographic monitoring, followed by the multiple sleep latency test, and showed that alterations in sleep quality and continuity were associated with hypersomnia. Age and dementia severity may also influence data from comparative studies, as can differences between testing methods. It was reported that CSF orexin-A levels were more sensitive and specific for the diagnosis of sleep disorders (in particular narcolepsy), and that orexin-A functions may be better investigated during CSF analyses (45). Also, damage to blood–brain barrier permeability could be a reason for increased orexin-A plasma levels in PD patients; however, whether plasma orexin-A levels reflect central orexin-A activity remains controversial.

Orexin-A was shown to interact with multiple regions of the brain stem and other neurotransmitter systems, including dopamine (46). The loss of dopamine neurons from the central amygdala decreased dopamine release in DLB and was related to hypersomnia in DLB. Patients with DLB exhibited an uptake reduction in ^123^I-FP-CIT SPECT, an imaging technology that measures extrastriatal serotonergic transporters in the amygdala. Also, a previous study reported that hypersomnia was associated with depression in DLB (47). Depression and hypersomnia in DLB may be driven by deficits in the noradrenergic system and impact arousal, cognition, and mood (48). Notably, dopamine agonists (DAs) increased hypersomnia or narcolepsy in DLB, though these therapeutics improved motor symptoms in patients with DLB. Therefore, the adverse effects of DAs on sleep in patients with LBD should be fully considered, particularly for patients with hypersomnia.

Currently, studies on CSF orexin-A levels in LBD are lacking. Additionally, different studies in this investigation were controversial. In terms of comparisons between LBD and AD, four studies were identified; hence, the trustworthiness of these results is questionable in this meta-analysis. In terms of the discrimination potential of orexin-A between LBD and FTLD patients, we identified only two studies; therefore, further studies are required before definitive conclusions can be made on the reliability of this biomarker.

## Conclusions

Our meta-analysis indicated that CSF orexin-A levels could function as a reliable biomarker to discriminate between LBD and AD/FTLD patients and potentially increase the accuracy of clinical diagnoses for these conditions. In the future, larger sample-size studies must be conducted to confirm and investigate the mechanisms underpinning our findings.

## Data Availability Statement

The original contributions presented in the study are included in the article. Further inquiries can be directed to the corresponding author.

## Author Contributions

JG conceived the research design. JG, ZC, and JH conducted literature search and data extraction. JG, X-DW, and LM performed the statistical analysis and contributed to writing of the report. SL and YJ reviewed and edited the article in detail. All authors contributed to the article and approved the submitted version.

## Funding

This work was supported by the National Natural Science Foundation (grant number 82171182), the National Key Research and Development Program of China (grant number 2016YFC1306305), Science and Technology Project of Tianjin Municipal Health and Health Committee (grant number ZC20121), and Science and Technology Project of Tianjin Municipal Health and Health Committee (grant number KJ20048).

## Conflict of Interest

The authors declare that the research was conducted in the absence of any commercial or financial relationships that could be construed as a potential conflict of interest.

## Publisher’s Note

All claims expressed in this article are solely those of the authors and do not necessarily represent those of their affiliated organizations, or those of the publisher, the editors and the reviewers. Any product that may be evaluated in this article, or claim that may be made by its manufacturer, is not guaranteed or endorsed by the publisher.
